# Anti-inflammatory effects of nicotinamide mononucleotide (NMN) in human skeletal muscle after BFR-exercise

**DOI:** 10.1080/15502783.2026.2632284

**Published:** 2026-02-18

**Authors:** Dai-Lin Yang, Kuo-Ching Chao, Hui-Tai Yang, Kuei-Hung Chen, Luthfia Dewi, Giancarlo Condello, Mengxin Ye, Andrew Nicholls, Yu-Chieh Liao, Chih-Yang Huang, Chia-Hua Kuo

**Affiliations:** a Laboratory of Exercise Biochemistry, University of Taipei, Taipei, Taiwan; b Department of Internal Medicine, China Medical University Hospital, Taipei, Taiwan; c Department of Nutrition, Universitas Muhammadiyah Semarang, Semarang, Indonesia; d Department of Medicine and Surgery, University of Parma, Parma, Italy; e College of Physical Education and Health Science, Zhejiang Normal University, Jinhua, China; f Department of Biotechnology, Asia University, Taichung, Taiwan; g Cardiovascular and Mitochondria Related Disease Research Center, Hualien Tzu Chi Hospital, Buddhist Tzu Chi Medical Foundation, Hualien, Taiwan; h Department of Medical Research, China Medical University Hospital, China Medical University, Taichung, Taiwan; i Department of Health and Physical Education, Laboratory of Exercise Biochemistry, The Education University of Hong Kong, New Territories, Hong Kong SAR; j School of Physical Education and Sports Science, Soochow University, Suzhou, China; k Department of Movement Sciences and Sports Training, School of Sport Sciences, University of Jordan, Amman, Jordan

**Keywords:** Ischemic reperfusion, p16^INK4A^, p21, inflammation, NETosis, mitochondrial transfer

## Abstract

**Background:**

β-Nicotinamide mononucleotide (NMN) inhibits acute inflammation in injured animal tissues.

**Aim:**

We examined whether NMN supplementation attenuates inflammation induced by blood flow restriction-resistance exercise (BFR-exercise) in human skeletal muscle.

**Methods:**

Eleven untrained men (22.8 ± 1.5 y) completed a randomized, placebo-controlled, counterbalanced crossover trial, receiving either Placebo or NMN (1200 mg/d) for 7 d, with a 3-week washout between conditions. Multiple muscle biopsies were obtained before and after BFR-exercise.

**Results:**

BFR-exercise-induced significant muscle necrosis at 0 h, which resolved within 24 h in both conditions. NMN supplementation suppressed exercise-induced increases in TNF-α and IL-10 mRNA but delayed the rise in p21 mRNA, suggesting attenuated inflammatory signaling and delayed myogenic differentiation. The resolution of infiltrating cells from necrotic regions was moderately delayed by NMN. BFR-exercise increased the mitochondrial content in exercised muscle by 171% after 24 h of recovery. However, this adaptation was abolished with NMN. Immunofluorescence staining with TOM20 and myeloperoxidase (MPO) revealed that infiltrating phagocytes carried substantially more mitochondria than myofiber cytoplasm, forming a diffusion gradient toward damaged regions of myofibers. This concentration difference between phagocytes and myofibers was further confirmed using COX4 immunostaining in biopsied muscle from an additional participant.

**Conclusions:**

NMN supplementation, while inhibiting inflammatory signaling in exercised human skeletal muscle, may also suppress mitochondrial replenishment from phagocytes to repairing myofibers.

## 1. Introduction

β-Nicotinamide mononucleotide (NMN) is a naturally occurring precursor to nicotinamide adenine dinucleotide (NAD+), a vital coenzyme that supports energy metabolism and DNA repair. It is enriched in plant foods like edamame, avocado, broccoli, and tomato [1]. NMN supplementation attenuated cell infiltration and neutrophil number in lipopolysaccharide (LPS)-induced acute lung injury in mice. The reversal of inflammatory cytokine expression [2] and the restoration of mitochondrial loss in TNF-α-treated cells *in vitro* [3] suggest a suppressive effect of NMN on inflammatory signaling. To date, the effects of NMN on exercise-induced inflammation and mitochondria quantity in human skeletal muscle have not been documented.

Resistance exercise is a muscle-damaging stressor that initiates significant inflammation. However, this process ultimately reduces cellular senescence [4] and promotes muscle hypertrophy [5]. The transformation of this potentially harmful stimulus into an adaptive benefit is mediated by immune cell infiltration into damaged regions of skeletal muscle [6]. In the early inflammation period (phagocytic phase), tumor necrosis factor-alpha (TNF-α) is released from immune cells to facilitate the death of unhealthy cells and debris removal [7]. This is followed by a release of interleukin-10 (IL-10), which downregulates phagocytic activity and promotes cell regeneration in repairing tissues [8]. The resolution speed of the entire inflammatory response reflects an individual's recovery capacity and, consequently, their overall fitness against challenges.

Modulating the inflammatory process directly impacts muscle adaptation. For instance, high doses of anti-inflammatory drugs hinder normal training adaptation in muscle hypertrophy and strength gains [9]. In contrast, acute proinflammatory supplements of a parasitic fungus powder before exercise accelerated the resolution of inflammation and myogenic differentiation in human skeletal muscle [10]. An acute pre-exercise blood flow restriction (BFR) amplifies the inflammatory response through ischemia-reperfusion injury [11], thereby further enhancing muscle hypertrophy induced by exercise [12]. However, unresolved or excessive immune cell infiltration can cause tissue damage [13] and bone marrow cell exhaustion [14]. The effects of NMN supplementation on inflammation in human skeletal muscle induced by BFR-exercise have not been reported.

Inflammation can also be triggered by aged or dysfunctional mitochondria [15]. In skeletal muscle, mitochondria have a half-life of approximately two weeks [16], and exercise stimulates mitophagy to remove these damaged organelles [17]. Timely replenishment of newly synthesized mitochondria is essential for maintaining low levels of inflammation in myofibers. Recent studies have identified immune cells originating from the bone marrow as mitochondrial-portable, capable of transferring mitochondria between cells [18,19]. Neutrophils, in particular, are a major immune cell carrying mitochondria, although their functional role remains unclear since these cells rely predominantly on glycolysis for ATP production [20].

In this study, we hypothesized that one week of NMN supplementation modulates the inflammatory response induced by blood flow restriction applied before acute, moderate-intensity resistance exercise (BFR exercise). Here, we examined mitochondrial abundance and cellular distribution in human skeletal muscle biopsies during the 24-h post-exercise recovery period. Additionally, we assessed whether NMN influences myogenic terminal differentiation during BFR-exercise recovery, as indicated by p21 mRNA expression in myocytes [21].

## 2. Materials and methods

### 2.1. Ethical approval

All participants volunteered to join in this study and signed the informed consent form after giving explanation of the whole experimental procedures and possible discomforts. This study was approved by the Institutional Review Board of the University of Taipei (IRB-2021-082) and conducted according to the Declaration of Helsinki.

### 2.2. Participants

The minimal sample size for this study was determined using G-Power software, assuming an effect size of |ρ| = 0.5, an alpha error probability of 0.2, and a power (1 − β error probability) of 0.8. A total of 15 healthy participants from Taipei city were initially enrolled in the study; however, 4 withdrew because of scheduling conflicts. The remaining 11 participants were young, non-athletic males with prior weight-training experience from physical education classes (age: 22.8 ± 1.5 y; height: 174.0 ± 6.4 cm; weight: 67.8 ± 12.8 kg). Inclusion criteria required participants to be male and between 20 and 30 y old. The exclusion criteria included recent muscle injury, the use of any medications, known allergies, chronic alcohol consumption, smoking, or specific metabolic conditions within the past two months. During the trial, participants were instructed to avoid exercise and the intake of nutritional supplements and anti-inflammatory drug unless directed otherwise by the research team. Familiarization with laboratory procedures was provided during the one-repetition maximum (1RM) squat test, unless otherwise specified in the experimental protocol.

### 2.3. Study design

To evaluate the effects of NMN supplementation on inflammation and mitochondria in skeletal muscle after BFR-exercise, a double-blind, placebo-controlled crossover study was conducted, with a 3-week washout period between conditions. The participants were randomly assigned in a balanced order to receive either NMN or a Placebo. Experimenter and participant were blind with supplements (assigned as supplements 1 and 2). NMN and Placebo capsules were identical in appearance, size, and color to minimize placebo effects. The experimenters and participants were blinded to the group assignments throughout the study.

During the first visit, a baseline muscle biopsy of the right vastus lateralis was obtained three weeks before the one-repetition maximum (1-RM) squat strength test. The BFR-exercise challenge was administered two weeks after the 1-RM test. Muscle biopsies were collected from the right leg immediately after the exercise challenge and from the corresponding location on the left leg 24 h later. Following a 4-week washout, participants crossed over to the alternate condition, and the protocol was repeated.

### 2.4. NMN supplementation

Participants received oral gelatin capsules containing 300 mg of NMN (AbinoNutra™ NMN, Shelton, Connecticut, USA) or 300 mg of cornstarch (Placebo), following protocols consistent with recent studies [22]. Capsules were taken four times daily—once after each of the three main meals and once before bedtime, beginning six days prior to the BFR-exercise challenge. This regimen provided a total daily dose of 1200 mg. The same protocol was maintained on the seventh day, which included the 24-h post-exercise recovery period.

To control for the potential influence of breakfast variation on post-exercise recovery, participants consumed a standardized liquid meal replacement one hour before the BFR-exercise (9:00–10:00 am): a can of Ensure® Original (Abbott Laboratories, Illinois, USA), providing 250 kcal (6 g fat, 34 g carbohydrates and 9 g protein), along with the supplement capsule in the morning before each muscle biopsy (pre-exercise baseline, 0 h and 24 h after exercise). Although this is not a typical meal for healthy individuals, the liquid formulation allows rapid delivery of nutrients and supplements prior to experimental challenge. Additionally, to minimize dietary variation after exercise, a standardized lunch was provided between 12:00 and 13:00 for each crossover trial (773 kcal total; 56% carbohydrate, 29% fat and 15% protein by energy).

### 2.5. Muscle strength test

The maximal lower-body strength was assessed using the one-repetition maximum (1-RM) Smith squat test, following the guidelines of the National Strength and Conditioning Association (NSCA). After a general warm-up consisting of dynamic stretching, the participants performed a specific warm-up sequence: one set of 10 repetitions at approximately 50% of the estimated 1-RM (roughly half of their body weight). After a 5-min rest, the participants completed 3–5 attempts with progressively heavier loads (increased by ~5% each attempt), with 3–5 min of rest between trials, until their true 1-RM was determined. The standardized squat technique was enforced: participants adopted a shoulder-width stance, performed a controlled descent, avoided bouncing at the bottom position, and maintained an upright torso with both feet flat on the floor throughout the movement.

### 2.6. Blood flow restriction (BFR)

Before the resistance exercise challenge, three episodes of BFR were applied with a standard sphygmomanometer and a cuff (CK-110; Spirit, New Taipei, Taiwan). Before BFR, the participants sat comfortably on the bed with their legs extended in a relaxed position. The blood pressure cuff was placed around the upper thighs just below the hip line. During BFR, the cuff was inflated to 180 mmHg for 5 min to occlude arterial inflow (total occlusion). This process was repeated three times for each limb, with a 5-min interval between each pressurization, followed by a 5-min period of rest after reperfusion (cuff release) at the same location. Five minutes following the last session of BFR, the participant performed a 5-min dynamic warm-up exercise before leg squatting. Each participant completed a set of warm-up exercises followed by a set of 8 repetitions (50% of 1-RM).

### 2.7. Exercise challenge

Resistance exercise was imposed to participants approximately 10 min after BFR (including the 5-min warm-up) at 10:00–11:00 am. The back squat exercise comprised 4 sets, each with 8 repetitions (70% of 1-RM), with a 90-s rest interval between sets on a Smith machine (Cybex, Minnesota, USA). It was recommended that participants perform squats by allowing their hips to touch an adjustable multi-angle fitness bench to reach a knee angle less than 90° (parallel to the ground). The participants' compliance was 100%. A trained supervisor monitored the exercise challenge programs for all the participants in the weight training room to ensure consistency of motion.

### 2.8. Rates of perceived exertion (RPE)

After each set of squats, the participants were instructed to immediately self-report their rating of perceived excretion (RPE). The participants reported the RPE by observing a numerical range from 1 “rest” to 10 “maximum effort.” [23].

### 2.9. Muscle biopsy

Muscle biopsy was always performed at 10:30 am to 11:30 pm at baseline, post-exercise (0 h), and recovery (24 h). Following a local anesthesia by lidocaine hydrochloride (Recipharm Monts, Monts, France), muscle samples were collected from the vastus lateralis muscle in depth of 3 cm from the skin surface using a 14 G Temno-TM biopsy needle (T149, CareFusion, Vernon Hills, Illinois, USA). To avoid potential inflammation caused by biopsy affecting the outcome measurements, baseline muscle biopsies were performed on the right leg 5 weeks before the squat challenge. Immediately after the squat exercise challenge, a muscle biopsy was conducted on the right leg, and 24 h later, a muscle biopsy was performed on the left leg. For each time point, 5–10 mg of muscle samples were collected through three needle insertions. Muscle samples for quantitative PCR analysis were immediately frozen on dry ice at the time of collection and then stored at −80 °C. Muscle samples for immunofluorescence staining were preserved in 4% paraformaldehyde immediately after muscle tissues removal.

### 2.10. Real-time polymerase chain reaction (RT-PCR)

Total RNA was extracted from approximately 5 mg of biopsied muscle using the RNeasy® Fibrous Tissue Mini Kit (QIAGEN, Hilden, Germany). Muscle samples were homogenized in 300 μl of Buffer RLT (containing guanidine thiocyanate to inhibit endogenous RNases) using a POLYTRON® homogenizer (PT 3100 D, KINEMATICA AG, Malters, Switzerland) on ice. The lysate was incubated at 55 °C for 10 min with 590 μl of RNase-free water and 10 μl of proteinase K solution to digest proteins and release nucleic acids.

RNA was isolated by binding to the silica membrane of the RNeasy Mini spin column. After adding 450 μl of 99.5% ethanol and centrifugation, DNase treatment was performed on the membrane for 15 min at room temperature to remove genomic DNA. The contaminants were washed off with Buffer RPE, and RNA was eluted in 30 μl of RNase-free water.

Reverse transcription was performed using 1 μg of total RNA and the iScript™ cDNA Synthesis Kit (Bio-Rad, Hercules, CA, USA), along with the TOOLS Quant II Fast RT Kit (TOOLS, New Taipei City, Taiwan), according to the respective manufacturers' protocols.

Quantitative RT-PCR was carried out in duplicate for each sample using the iTaq Universal Probes Supermix (Bio-Rad) and gene-specific primers and probes. The target genes were amplified using PrimePCR™ probe assays (Bio-Rad) or TaqMan® assays (Thermo Fisher Scientific, MA, USA) with the following assay IDs: RPP30 (Hs01124518_m1), TNF-α (Hs00174128_m1), IL-10 (Hs00961622_m1), and p21 (Hs00355782_m1). Reactions were run on a CFX96 Touch Real-Time PCR Detection System (Bio-Rad) using the standard protocol: initial denaturation at 95 °C for 30 s, followed by 40 cycles of 95 °C for 5 s and 55 °C for 30 s.

Gene expression was normalized to the geometric mean of the housekeeping gene RPP30 and calculated using the 2^−ΔΔCt^ method, expressed as a fold change relative to RPP30.

### 2.11. Immunofluorescence staining

Following muscle sample collection, approximately 5 mg of each muscle slice was immediately fixed in 1.5 μL of 4% paraformaldehyde and sent to the Biotechnology Center (Toson Technology, Hsinchu, Taiwan) for immunofluorescence analysis targeting the nucleus, TOM20, MPO, and p16^INK4a^ antibodies.

Immunofluorescence image quantification followed our previously established procedures [24]. In this crossover study, muscle samples collected at baseline, after exercise, and during NMN supplementation from each participant were sectioned and mounted on the same glass slide to minimize staining variability.

Tissue sections were first pretreated with citrate buffer (pH 6.0) for 12 min at room temperature. After pretreatment, an immunoblocking reagent containing multiple IgG-binding proteins, ions, and a chelating agent (BioTnA, Kaohsiung, Taiwan) was applied for 30 min at room temperature. The first primary antibody, rabbit anti-human p16^INK4a^ (ab108349, 1:500, Abcam, Cambridge, UK), was then incubated for 16 h at room temperature. Detection was performed using a secondary antibody, goat anti-rabbit IgG (H+L)-488 (TAFB02-488, BioTnA, Kaohsiung, Taiwan), for 30 min, producing a green fluorescence signal.

After another round of immunoblocking (60 min, room temperature), the second primary antibody, rabbit anti-human MPO (ab9535, 1:100, Abcam, Cambridge, UK) was applied and the samples were incubated for 16 h at room temperature. Detection was done using goat anti-rabbit IgG (H+L)-594 (TAFB02-594, BioTnA, Kaohsiung, Taiwan) for 30 min, resulting in red fluorescence.

Following a third immunoblocking step (60 min), the third primary antibody, rabbit anti-human TOM20 (GTX133756, 1:1000, GeneTex, California, USA), was incubated for 1 h at room temperature. Detection was achieved using goat anti-rabbit IgG-iFluor-680 (TAFB02-680, BioTnA, Kaohsiung, Taiwan) for 30 min, yielding a pink fluorescence signal.

For nucleated cell infiltration, the nuclei were counterstained with 4′,6-diamidino-2-phenylindole (DAPI) (TA01DP, BioTnA, Kaohsiung, Taiwan).

Immunofluorescence staining images from the microscope was visualized by OlyVIA Ver.2.9.1 software (Olympus Europa, Hamburg, Germany) and analyzed by Image J Version 1.53t (National Institutes of Health, Maryland, USA). The quantitative criteria for cell infiltration are that over 7 nuclei aggregated together in cytoplasm and the quantitative criteria for centrally located nuclei are those nuclei inside myofibers.

### 2.12. Statistical analysis

All the data are presented as mean ± standard error (SE). Statistical analyses were conducted using SPSS version 27.0.1.0 (IBM, Armonk, NY, USA). The primary outcomes are cell infiltration and the number of mitochondria in muscle tissues. Paired *t*-tests were used to compare differences between baseline and post-exercise, as well as to assess the effects of NMN at 24 h post-exercise against Placebo. A Type I error rate of ≤5% (*p* ≤ 0.05) was considered statistically significant when comparing the mean differences. Effect sizes were calculated using Cohen's *d* and interpreted as small (<0.50), medium (0.50–0.79), and large effect (≥0.80).

## 3. Results

### 3.1. Participants

**Figure 1.**
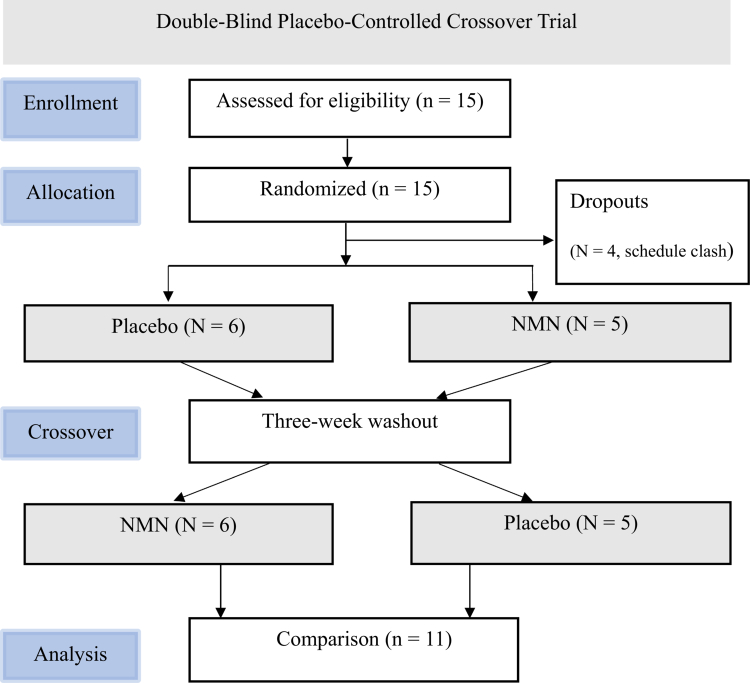
Flow chart of the study.

The causal effects of NMN on inflammation and mitochondrial function in skeletal muscle were examined in 11 young men after excluding 4 participants due to scheduling conflicts (Figure 1).

### 3.2. Inflammatory cytokine expression

**Figure 2.**
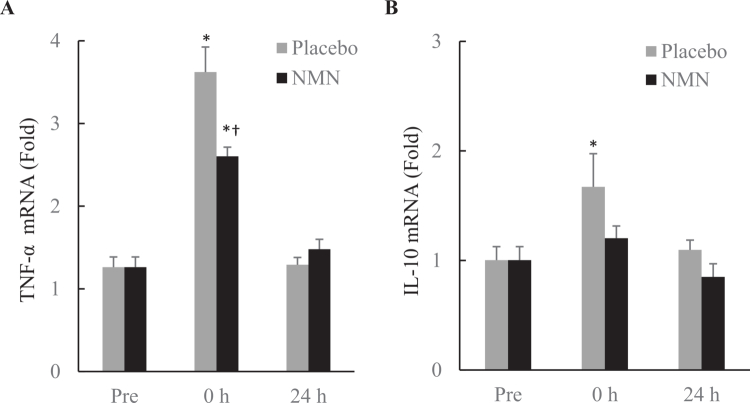
Inflammatory cytokine mRNA expression in human skeletal muscle after BFR-exercise. Both TNF-α mRNA (A) and IL-10 mRNA (B) increased immediately after BFR-exercise. However, the TNF-α mRNA response was attenuated and the IL-10 mRNA response was minimized in NMN supplemented condition. *Significant difference against baseline, *p* < 0.05. ^†^Significant difference against Placebo at the same time point, *p* < 0.05. Abbreviations: blood flow restriction-preconditioned resistance exercise (BFR-exercise) and NMN: nicotinamide mononucleotide.

Rating of perceived exertion (RPE) was self-reported by participants at the end of BFR-exercise. RPE was 8.5 ± 0.3 arbitrary units (AU) in the Placebo-supplemented condition and 8.1 ± 0.4 AU in the NMN supplemented condition. NMN supplementation did not significantly alter the RPE. BFR-exercise transiently increased the mRNA expression of inflammatory cytokines in human skeletal muscle (Figure 2). Tumor necrosis factor alpha (TNF-α) increased by 187% (*p* < 0.01, *d* = 1.58) and returned to baseline within 24 h (Figure 2A). This acute response was attenuated by NMN supplementation, showing a smaller increase of 106% (*p* < 0.01, *d* = 2.31). Interleukin-10 (IL-10) also increased by 67% immediately after BFR-exercise (*p* < 0.05, *d* = 0.86) and normalizing after 24 h (Figure 2B), while no significant change in IL-10 was observed with NMN supplementation.

### 3.3. Nucleated cell infiltration

**Figure 3.**
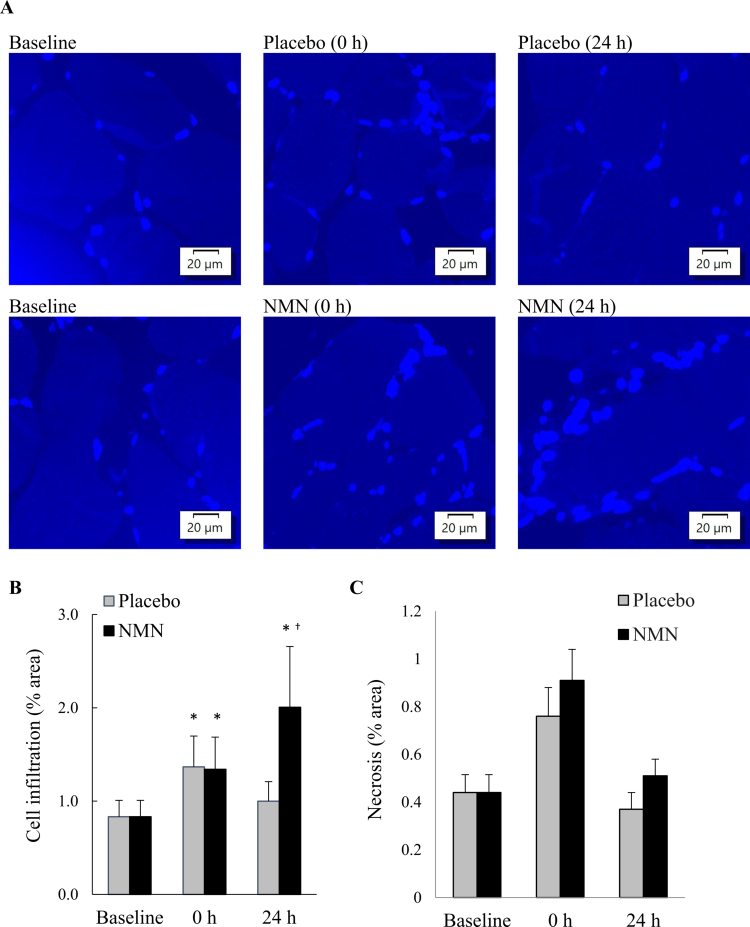
Nucleated cell infiltration in human skeletal muscle after BFR-exercise. Representative image of DAPI-stained muscle tissue showing nucleated cell infiltration (A). BFR-exercise-induced nucleated cell infiltration in muscle tissues was completely attenuated by 1-week NMN consumption (*n* = 11), (B). Hematoxylin and eosin (H&E) staining shows no significant difference in necrosis area between the Placebo and NMN and (C). *Significant difference against baseline, *p* < 0.05. ^†^Significant difference against Placebo group at the same time point, *p* < 0.05. Abbreviations: blood flow restriction-preconditioned resistance exercise (BFR-exercise) and NMN: nicotinamide mononucleotide.

Nucleated cell infiltration in skeletal muscle was quantified by DAPI staining, which was defined as the percentage of necrotic muscle area containing aggregated nuclei (≥7 nuclei per region) relative to the total myofiber area (Figure 3). BFR-exercise-induced a moderate increase in cell infiltration (+65%, *p* = 0.09, *d* = 0.59), which returned to baseline after 24 h. Under NMN supplementation, cell infiltration continued to elevate 24 h after BFR-exercise (+142%, *p* < 0.05, *d* = 0.71).

### 3.4. Mitochondrial diffusion from neutrophils to myofibers

**Figure 4.**
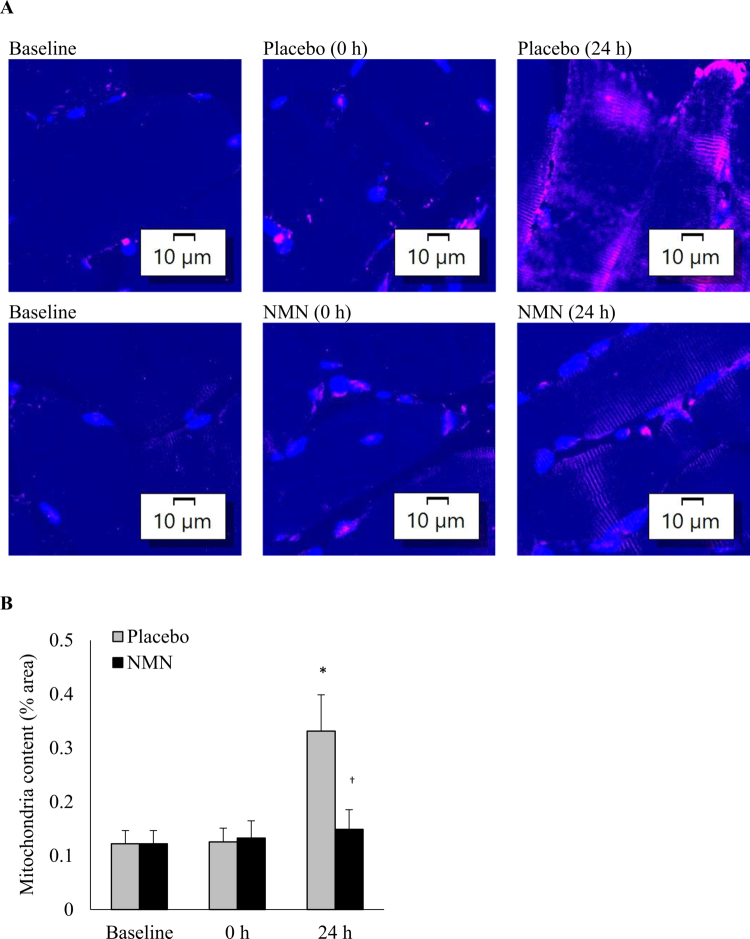
Mitochondrial content in human skeletal muscle after BFR-exercise. Representative immunofluorescence-stained images show the mitochondrial content (labeled by TOM20 antibody) in muscle tissues (A). BFR-exercise-induced a significant increase in the mitochondrial content in muscle tissues, but this response was blocked in NMN-supplemented condition. *Significant difference against baseline, *p* < 0.05. ^†^Significant difference against Placebo at the time point, *p* < 0.05. Abbreviations: blood flow restriction-preconditioned resistance exercise (BFR-exercise) and NMN: nicotinamide mononucleotide.

Immunofluorescence staining labeled by TOM20 provides direct assessment of the mitochondrial content in muscle tissues (Figure 4). The mitochondrial content in muscle tissues substantially increased 24 h after BFR-exercise (+171%, *p* = 0.02, *d* = 2.60) and this exercise response was prevented by NMN supplementation.

**Figure 5.**
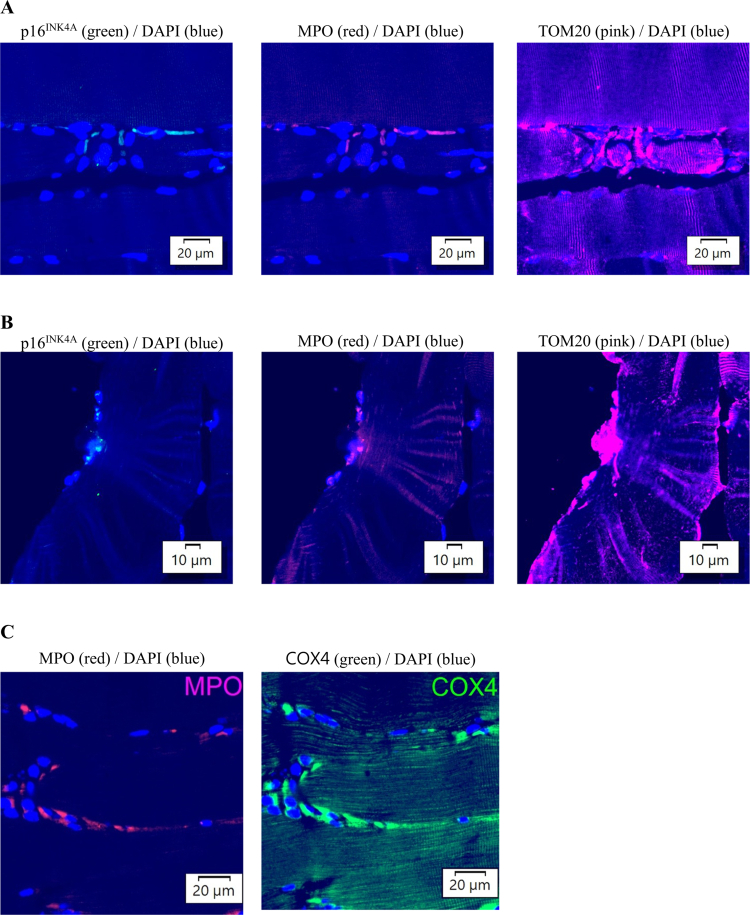
Mitochondria diffusion from neutrophils to engaged damaged myofiber. Representative immunofluorescence-stained images of muscle cross-section of a young man, demonstrating that neutrophils engaging to myofibers at 0 h post-BFR-exercise (A) and that myofiber-engaged neutrophils at 24 h post-BFR-exercise (B). Neutrophils are labeled by myeloperoxidase (MPO) antibody (red fluorescence). The mitochondria are labeled by TOM20 antibody (pink fluorescence). Substantially higher mitochondrial content in neutrophils outside myofibers is reconfirmed by immunostaining by COX4 antibody (green) (C). All the neutrophils appear colocalized with the cellular senescence marker p16^INK4a^ (green fluorescence) along the extramyofibrillar boundary of the myofibers in the necrotic zone. Abbreviations: blood flow restriction-preconditioned resistance exercise (BFR-exercise) and NMN: nicotinamide mononucleotide; DAPI: 4′,6-diamidino-2-phenylindole.

While myofibers account for over 90% of the muscle cross-sectional area, the central cytoplasmic region of the myofibers exhibited the lowest mitochondrial density. The mitochondria were densely localized outside myofibers, particularly in necrotic regions, in both the resting and exercised muscle (Figure 4). To evaluate mitochondrial contributions from neutrophils, cells were labeled using myeloperoxidase (MPO; red) and co-stained with TOM20 (pink) and the cellular senescence marker p16^INK4a^ (green) in exercised tissues (Figure 5). Myofiber-engaged neutrophils exhibited a high mitochondrial concentration, which gradually decreased toward the cytoplasmic core region of myofibers. The highest mitochondrial densities were observed near disrupted myofibers within necrotic zones, suggesting that mitochondrial replenishment via immune response-mediated transfer occurs in the 24 h following exercise.

All the neutrophils were colocalized with p16^INK4a^ along the extramyofibrillar boundary of the myofibers. p16^INK4a^ expression is largely restricted to cells with mitotic potential, where it functions to regulate cell cycle arrest during senescence. Neutrophils are terminally differentiated, short-lived cells that do not enter the cell cycle. Thus, they are not known to express significant levels of p16^INK4a^. When imaging shows the colocalization of neutrophils (i.e. MPO^+^ cells) with p16^INK4a^, it is likely p16^INK4a^ is being expressed by adjacent senescent cells (e.g. endothelial cells, myocytes or interstitial fibroblasts). Neutrophils are known to accumulate near senescent cells due to a senescence-associated secretory phenotype (SASP) [25].

### 3.5. p21 mRNA expression

**Figure 6.**
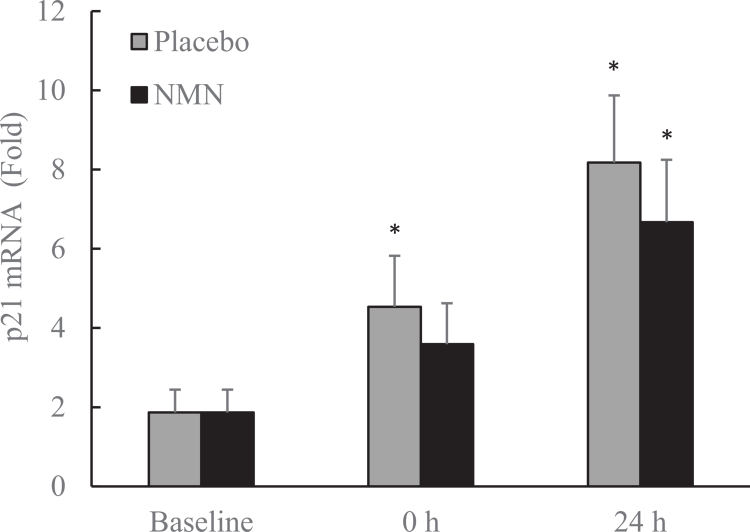
p21 mRNA in human skeletal muscle after blood flow restriction-preconditioned resistance exercise (BFR-exercise). p21 mRNA is a marker of cell-cycle arrest associated with myogenic differentiation. *Significant difference against baseline, *p* < 0.05. Abbreviations: Blood flow restriction-preconditioned resistance exercise (BFR-exercise) and NMN: nicotinamide mononucleotide.

Figure 6 illustrates the expression of p21 mRNA, a cell cycle inhibitor associated with terminal differentiation of myocytes [21]. BFR-exercise significantly increased p21 mRNA expression in skeletal muscle (+143%, *p* < 0.05, *d* = 0.89), with further elevation observed at 24 h post-exercise (+338%, *p* < 0.05, *d* = 1.66). NMN supplementation delayed this response, showing a lower increase at 24 h (+257%, *p* < 0.05, *d* = 1.35).

## 4. Discussion

### 4.1. Key findings

Inflammation, a local immune response, is a healing process essential for rejuvenating muscle tissue [26,27] and driving muscle growth following acute injurious challenges [8]. In this study, we employed pre-exercise BFR to elicit a robust inflammatory response in human skeletal muscle. The major findings are as follows: (1) NMN supplementation attenuates inflammatory mediator expression (TNF-α and IL-10 mRNA), delays nucleated cell infiltration and moderately suppresses p21 mRNA expression in skeletal muscle following BFR-exercise; (2) the mitochondrial concentration is substantially higher in nucleated cells surrounding myofibers than in those within the myofiber cytoplasm. In particular, directional mitochondrial donation from infiltrating neutrophils to damaged myofibers occurs in disruption sites and (3) NMN supplementation prevents BFR-exercise-induced increases in the mitochondrial content within muscle tissue. Taken together, these findings reveal a novel perspective on the contribution of mitochondria-enriched immune cells to muscle regeneration following intense physical activity.

### 4.2. NMN suppresses inflammatory cytokine expression in human skeletal muscle after BFR-exercise

This is the first human study reporting the suppressive action of NMN supplementation on inflammatory cytokine mRNA expression (i.e. TNF-α and IL-10) in skeletal muscle against BFR-exercise-induced damage. Inflammation is essential for muscle growth after injurious challenges [8]. During the immune response, TNF-α levels rise in the phagocytic phase to guide the clearance of senescent cells and cellular debris by neutrophils (MPO-expressing cells), followed by an increase in IL-10 during the regenerative phase to support muscle tissue renewal and close phagocytosis [28]. The rise-and-fall of cytokines orchestrates the complete tissue repair process. The observed suppression of TNF-α and IL-10 expression demonstrates an interference to immune response from NMN supplementation, consistent with findings reported from animal studies in tissue damage models [29,30].

### 4.3. NMN supplementation delays recovery in BFR-exercise-induced cell infiltration

Blood-borne cells originating from the bone marrow are key players in wound healing [31]. During muscle inflammation, a diverse range of myeloid cells presents in human skeletal muscle [8]. The suppressive effects of NMN on the inflammatory signaling system appear to delay the resolution of nucleated cell infiltration in skeletal muscle following BFR-exercise. In this study, the type of invading cell population remains unclear. Previous studies have shown that anti-inflammatory interventions can reduce stem cell homing to skeletal muscle after exercise [32], whereas proinflammatory stimulation enhances stem cell recruitment and accelerates the resolution of inflammation [10]. The identification of the cell types infiltrated into challenged skeletal muscle influenced by NMN supplementation and responsible for muscle recovery requires further investigation.

### 4.4. NMN inhibits mitochondrial gains after BFR-exercise

BFR-exercise is known to stimulate mitochondrial gains in skeletal muscle [33]. Here, we have found an impeded mitochondrial gain in BFR-exercised human muscle tissue following NMN supplementation. Furthermore, we identified a high concentration of mitochondria in the cells surrounding myofibers, along with their diffusion pattern into the interior of the myofiber, suggesting a continuous replenishment from extramyofibrillar mitochondrial transfer. Emerging *in vitro* evidence suggests that many myeloid cells are mitochondrial carriers [18,19], capable of transferring mitochondria between cells [34].

Neutrophils are among the most abundant immune cell types essential for tissue repair and wound healing [35] and are frequently present in damaged tissues [18]. In this study, we have shown that neutrophils are mitochondria-enriched cells, despite their minimal role in oxidative phosphorylation for ATP production for the neutrophils themselves [20]. Our finding implicates an important additional role of neutrophils as a source for mitochondrial replenishment in damaged myofibers. This mitochondrial rejuvenation may explain the reduced mitochondrial ROS emissions, which signify younger mitochondrial population, in human skeletal muscle after BFR-exercise as previously reported [36]. The underlying mechanism explaining the inhibitory effect of NMN on mitochondrial gain requires further investigation.

### 4.5. NETosis-like mitochondrial delivery to damaged myofibers

Mitochondria age rapidly, with a relatively short lifespan of approximately 8–23 d [37]. Therefore, it is crucial to replenish fresh mitochondria to maintain youthful populations inside host cells [38]. In some damaged of myofibers, we found that mitochondria spread from myofiber-engaged neutrophils by forming web-like structures resembling neutrophil extracellular traps (NETs). Neutrophilic NETosis is commonly observed in compromised host cells infected by pathogens such as bacteria, fungi, and viruses [39]. This observation, along with evidence of projecting mitochondria from myofiber-engaged neutrophils to disrupted myofibers following BFR-exercise, suggests a new function of neutrophils in rejuvenating post-exercise muscle.

In human skeletal muscle, myofibers occupy more than 90% of the tissue area. Notably, in this study, these myofibers contain a relatively low concentration of mitochondria Compared to the engaged neutrophils outside the myofibrillar space (Figure 4). Previous studies have shown that increased mitochondria in human muscle can occur as fast as 1 h post-exercise [40], far exceeding the rate of mitochondrial doubling time of approximately 1–3 weeks [41]. *De novo* mitochondrial biogenesis inside myofibers cannot explain observed fast replenishment after exercise. The evidence of mitochondrial donation from invading neutrophils occurring within a short period provides a more reasonable explanation for fast adaptation that can occur within 1 d.

### 4.6. Limitation

Mitochondrial gains are generally considered as a favorable adaptation after exercise training, as more mitochondria allow efficient ATP production in cells. However, we could not conclude whether the lack of mitochondrial increase in BFR-exercised muscle by NMN supplementation should be considered a malignant metabolic outcome for humans. Decreasing the mitochondrial content in skeletal muscle by genetic manipulation (Surf1^loxP^) substantially increases longevity in mice [42]. The balance between the preservation of quiescent stem cells (low mitochondria, high cell renewal) in the bone marrow niche and activated stem cells (high mitochondria, mobilizable) for tissue regeneration may be more important to function and health *in vivo* [43]. Increasing mitochondria number can suppress stem cell self-renewal but promote differentiation [44]. In the present study, NMN was supplemented 4 times a day (3 meals before bedtime) for 6 d before and 1 d after the BFR-exercise. The question of whether separating NMN supplementation time 12 h before and after BFR-exercise time can produce a more metabolically balanced condition favoring long-term human health remains to be tested.

## 5. Conclusion

This study provides new evidence that NMN supplementation suppresses inflammatory signaling in human skeletal muscle induced by BFR exercise. We also observed that phagocytes (MPO-expressing cells) contained substantially more mitochondria than myofibers within muscle tissue. These phagocytes preferentially accumulated in disrupted regions, suggesting a damage-induced transfer of mitochondria from bone marrow–derived immune cells to challenged human skeletal myofibers. Notably, NMN supplementation also inhibited mitochondrial gains following BFR exercise. These findings suggest that the anti-inflammatory action of NMN interferes with mitochondrial transfer from phagocytes to stressed myofibers. Further research is warranted to determine whether optimizing the timing of NMN supplementation could enhance muscle adaptation to BFR exercise.

## Data Availability

The data that support the findings of this study are available on request from the corresponding author. Readers may contact Prof. Chia-Hua Kuo at chkuo@eduhk.hk to request the data.
